# Clinical evaluation of the efficacy of a new bone cement-injectable cannulated pedicle screw in the treatment of spondylolysis-type lumbar spondylolisthesis with osteoporosis: a retrospective study

**DOI:** 10.1186/s12891-022-05904-7

**Published:** 2022-11-03

**Authors:** Lei Song, Jun Xiao, Rui Zhou, Cong-can Li, Ting-ting Zheng, Fei Dai

**Affiliations:** 1 Department of Orthopaedics, First Affiliated Hospital, Army Medical University, Chongqing, 400038 People’s Republic of China; 2 Department of Special Service Physiological Training, Guangzhou Special Service Recuperation Center of PLA Rocket Force, Guangzhou, 515515 People’s Republic of China

**Keywords:** CICPS, Osteoporosis, Spondylolysis-type lumbar spondylolisthesis, Clinical evaluation

## Abstract

**Purpose:**

To investigate the clinical efficacy and safety of a bone cement-injectable cannulated pedicle screw (CICPS) in the treatment of spondylolysis-type lumbar spondylolisthesis with osteoporosis.

**Methods:**

A retrospective study was conducted on 37 patients (Dual-energy X-ray bone density detection showed different degrees of osteoporosis) with spondylolysis-type lumbar spondylolisthesis who underwent lumbar spondylolisthesis reduction and fusion using a new type of injectable bone cement screw from May 2011 to March 2015. Postoperative clinical efficacy was evaluated by the Visual Analogue Scale (VAS) scores and the Oswestry Disability Index (ODI). Imaging indexes were used to evaluate the stability of internal fixation of the devices 1, 3, 6, and 12 months after surgery and annually thereafter. The safety of the CICPS was assessed by the prevalence of intraoperative and postoperative complications.

**Results:**

A total of 124 CICPS were implanted intraoperatively. Bone cement leakage occurred in 3 screws (2.42%), and no clinical discomfort was found in any patients. All 37 patients were followed up with an average follow-up time of 26.6 ± 13.4 months (12–58 months). In the evaluation of the clinical effects of the operation, the average postoperative VAS score of the patients decreased from 4.30 ± 1.58 before surgery to 0.30 ± 0.70 after surgery (*P* < 0.001), and the ODI decreased from 47.27% ± 16.97% before surgery to 3.36% ± 5.70% after surgery (*P* < 0.001). No screw was loose, broken or pulled out.

**Conclusion:**

CICPS is safe and effective in the treatment of spondylolysis-type lumbar spondylolisthesis complicated by osteoporosis.

## Background

**Fig. 1:**
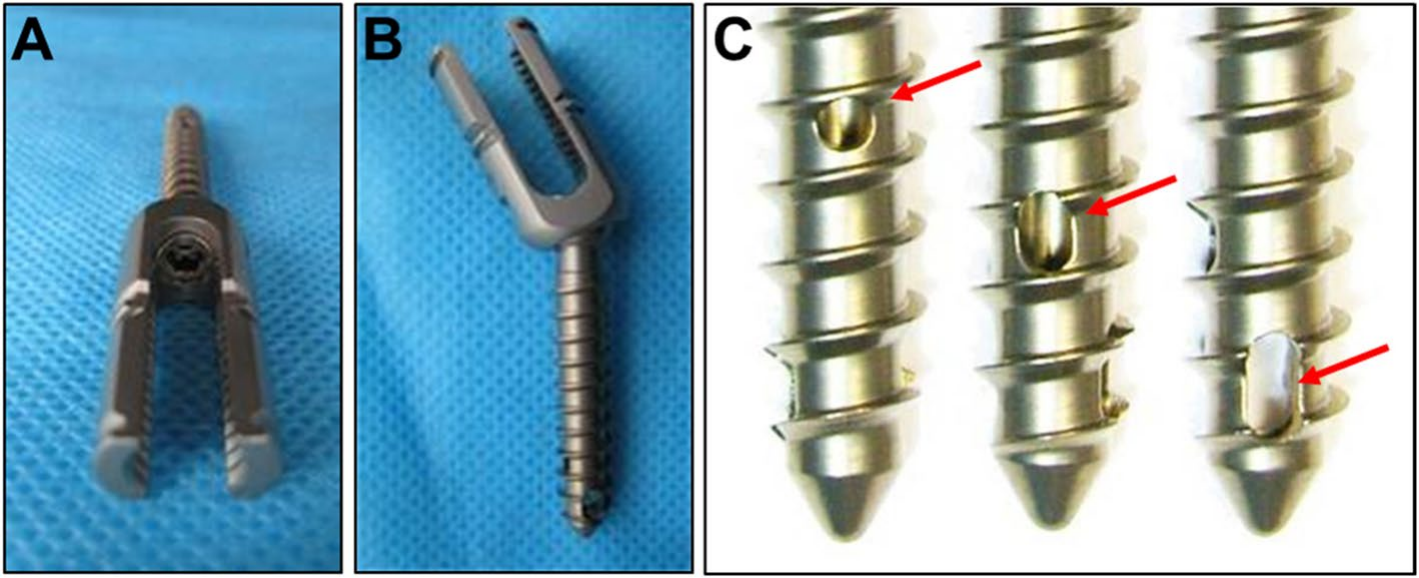
Gross observation of CICPS. **A** Top view of CICPS. **B** Side view of CICPS. **C** Three side holes distributed longitudinally from small to large

Lumbar spondylolisthesis is a common disease that causes chronic lumbar and leg pain in middle-aged and older people. The Wiltse Classification System classified lumbar spondylolisthesis into 5 types, including dysplasia, spondylolysis, degeneration, trauma and pathology, among which spondylolysis has the highest incidence [1, 2]. The main surgical treatment for spondylolysis-type lumbar spondylolisthesis is pedicle screw system. However, the postoperative screw loosening rate is extremely high in lumbar spondylolysis-type slippage patients with osteoporosis, due to the reduced bone mineral density, bone microarchitecture, and thinning of cortical bone. As Galbusera et al. reported that spondylolisthesis patients with osteoporosis had a screw loosening rate of 60% [3].To overcome screw loosening, cannulated pedicle screw (CPS) augmented by poly-methyl-methacrylate (PMMA) has become the most used method in spinal diseases with osteoporosis [4]. In our previous study, we designed a new type of pedicle screw named bone cement-injectable cannulated pedicle screw (CICPS) as shown in Fig. 1. Biomechanical test results proved CICPS had greater torque than the OMEGA cannulated pedicle screw and conventional pedicle screw, and finite element analysis showed none excessive stress at the screw-cement–bone interface in the CICPS group [5]. Afterwards, we have applied our CICPS in relevant clinical applications [6], all of which achieved satisfactory outcomes, and obtained national patent.

In this study, we retrospectively reviewed the preoperative and postoperative clinical manifestations and imaging parameters of 37 consecutive patients with spondylolysis-type lumbar spondylolisthesis complicated by osteoporosis using CICPS. The incidence of complications and the clinical efficacy of our CICPS were summarized.

## Materials and methods

### General data

From May 17, 2011 to March 12, 2015, clinical data of 37 patients with spondylolysis-type lumbar spondylolisthesis and osteoporosis were reviewed in this study. Inclusion criteria were as follows: 1. Confirmed diagnoses of spondylolysis-type lumbar spondylolisthesis by positive lumbar X-ray and plain lumbar Computed Tomography (CT) results with related clinical symptoms; 2. Confirmed diagnoses of osteoporosis by preoperative dual-energy X-ray bone density absorptiometry, T-score < -2.5 SD [7]; and 3. CICPS were used with no surgical contraindications. Exclusion criteria were as follows: 1. Allergy to the implant; 2. Presence of other spine diseases; and 3. Infections, blood system-related diseases, or other surgical contraindications. General information of patients was listed in Table 1. The study was approved by the Southwest Hospital ethics committee (KY201927), and carried out in accordance with ethical guidelines of Army Medical University. All patients included in this study gave their informed consents.

**Table 1:** Baseline demographic and clinical characteristics of 37 patients

Variable	Value
Mean age, years; mean ± SD (range)	60.11 ± 7.83(46 ~ 76)
Gender (n; M:F)	6:31
Mean BMD, T-score; mean ± SD (range)	-3.16 ± 0.59 (-2.5 ~ -5.0)
Area of spondylolisthesis (L4:L5)	17:20
Meyerding classification of spondylolysis	
I	10 (27.0%)
II	20 (54.1%)
III	6 (16.2%)
IV	1 (2.7%)

### CICPS design

CICPS (produced by Kanghui Medical Devices, Jiangsu, China) used in this study had diameters of 5.0 mm to 6.0 mm, lengths of 45 to 50 mm, nail heads adopting unidirectional and universal designs, unidirectional heads that can move with 360° rotation, and a longitudinal hollow center. The bone cement outflow diameter was 2.2 mm and was closed distally. Three side holes were designed in the front 2/5 of the screw and were distributed longitudinally from small to large. The side holes near the nail head were round with a diameter of 2 mm. The opposite side was separated by a thread with an oval side hole, a long diameter of 3 mm and a short diameter of 2 mm. The u-shaped side hole with a penetrating tip was 4 mm long and 2 mm wide (Fig. 1).

### Surgical method

**Fig. 2:**
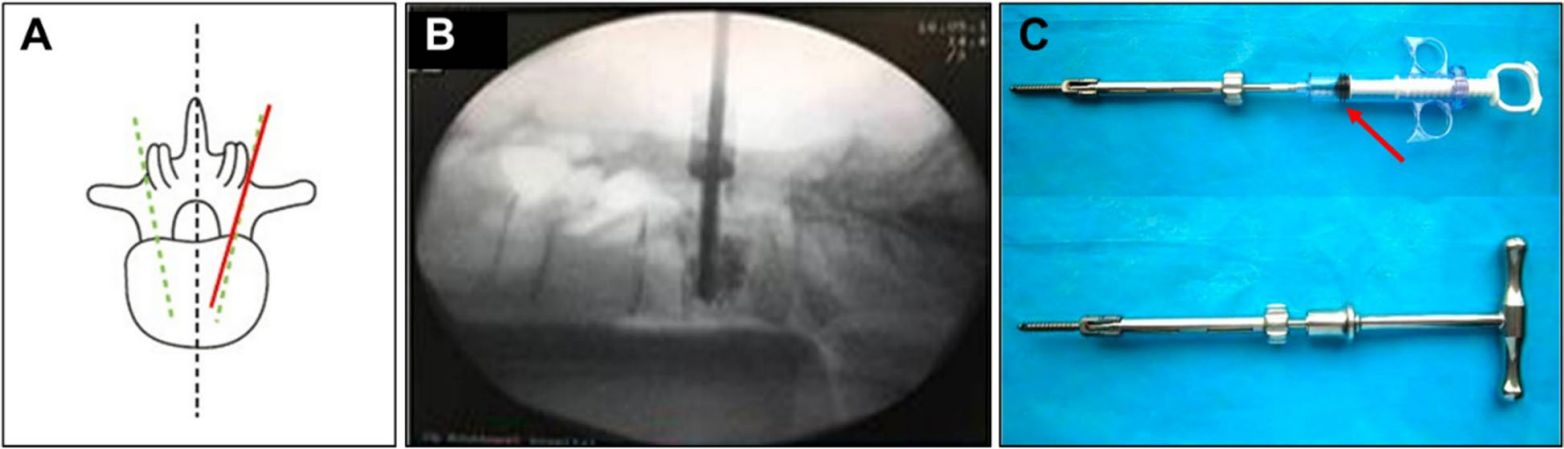
Surgical procedure of CICPS. **A** CICPS nailing angle slightly larger than normal pedicle screws, red line represents CICPS nailing angle and green line represents normal screws nailing angle. **B** Intraoperative bone cement dispersion under c-arm fluoroscopy monitoring. **C** Customized injection system of CICPS, red arrow indicates negative pressure aspirator

Transforminal lumbar interbody fusion was routinely performed. All the cases underwent vertebrae fusion, and whether single or double vertebrae were used depending on the condition of patients, all the cages used were purchased from Johnson & Johnson (USA). The surgical procedure used a conventional posterior midline approach with a nailing method similar to that of ordinary pedicle screws and a nailing angle slightly larger than for normal pedicle screws (Fig. 2A). To ensure that the screw hole side was away from the walls, the screw was placed 80% to 90% into the vertebra from the front wall, far away from the paries posterior of the vertebral body, to avoid leakage of bone cement into the spinal canal. Before nailing, a probe was used to ensure the integrity of the pin track was not damaged. Under c-arm fluoroscopy monitoring (Fig. 2B), bone cement in "toothpaste" period was injected through a customized injection system (Fig. 2C). Before bone cement injection, a negative pressure aspirator (Fig. 2C at the red arrow) was used to ensure the side hole of screw is not blocked by bone chips, otherwise, negative pressure should be repeatedly applied or the screws should be rotated to ensure an unobstructed side hole.

**Fig. 3:**
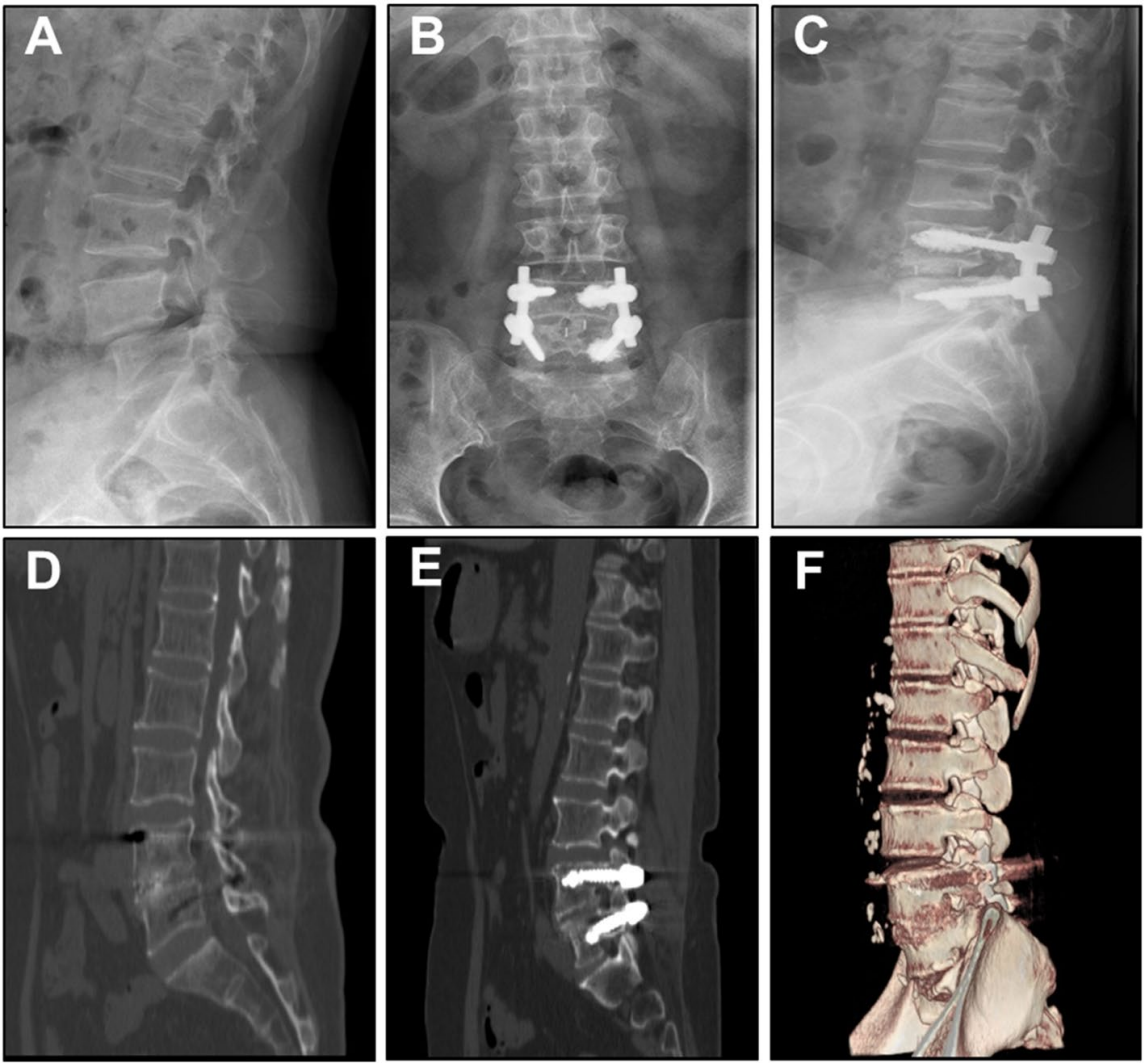
A 59 years old female, BMD -2.7SD, treated with CICPS fixation by PMMA augmentation. **A** Preoperative radiographs showed L4 vertebral body was of level II forward slip, spondylolysis type. **B** and **C** No bone cement leakage was observed one week after surgery. **D** CT results indicated successful fusion six months after surgery that continuous bone callus passed in the intervertebral space of fusion. **E** and **F** 56 months after the operation, CT showed good intervertebral fusion, no loosening or pulling out of screw was observed

**Fig. 4:**
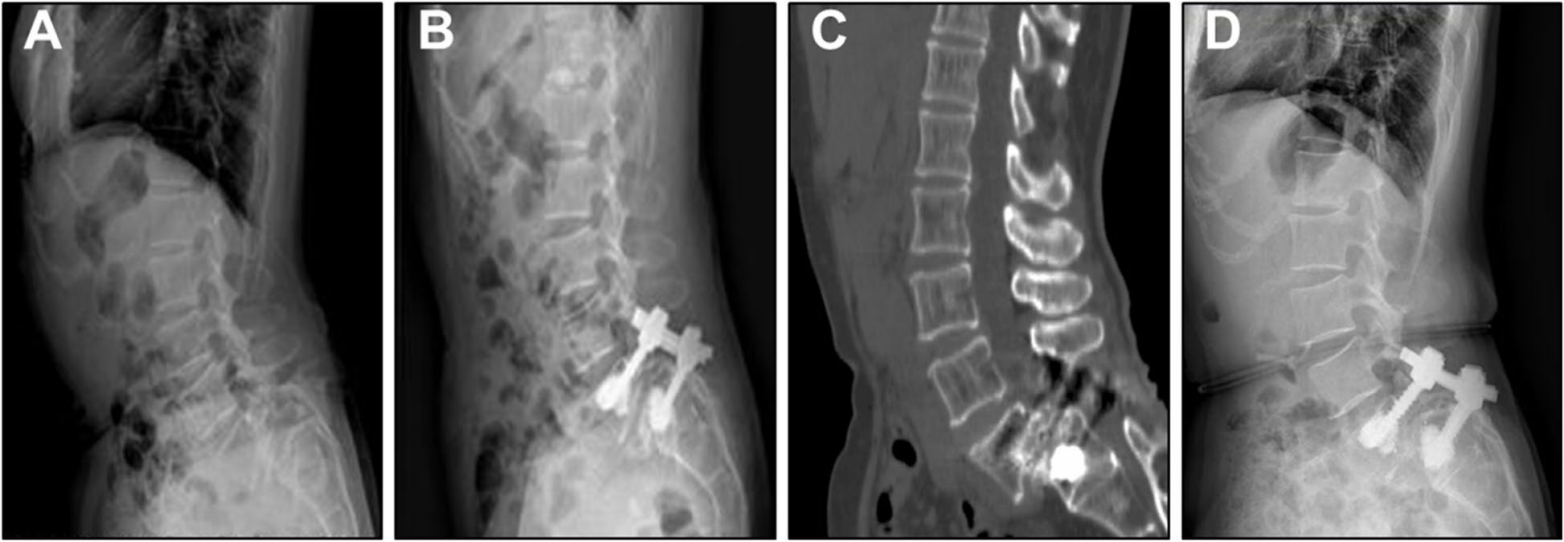
A 54 years old female, BMD -2.8SD, treated with CICPS fixation by PMMA augmentation. **A** Preoperative radiographs showed L5 vertebral body was of level IV forward slip, spondylolysis type. **B** X-ray examination showed good vertebral reduction and no leakage of bone cement one week after operation. **C** CT results showed good fusion 36 months after surgery. **D** 60 months after surgery, no obvious double ring sign, no loosening, no fracture or pulling out was observed

Considering the optimal strengthening effect need 1–2 ml of bone cement [6], and 1.5 ml of bone cement will remain in the supporting injection system, a total of 3 ml bone cement was injected during the operation. Decompression was performed on the spondylolisthesis, and the crushed autologous bone was filled into cages of appropriate size for bone fusion. After the bone cement was solidified, the connecting rod was bended and the sliding vertebral body was lifted for resetting (Figs. 3 and 4 for typical cases).

In order to save medical costs, common pedicle screw internal fixation was given because the surgeon felt that the bone of some patients was fine during the operation. All patients were given anti-osteoporosis therapy, including calcium/vitamin D supplementation, aluminum phosphate or zoledronic acid salt according to the degree of osteoporosis.

### Evaluation method

**Fig. 5:**
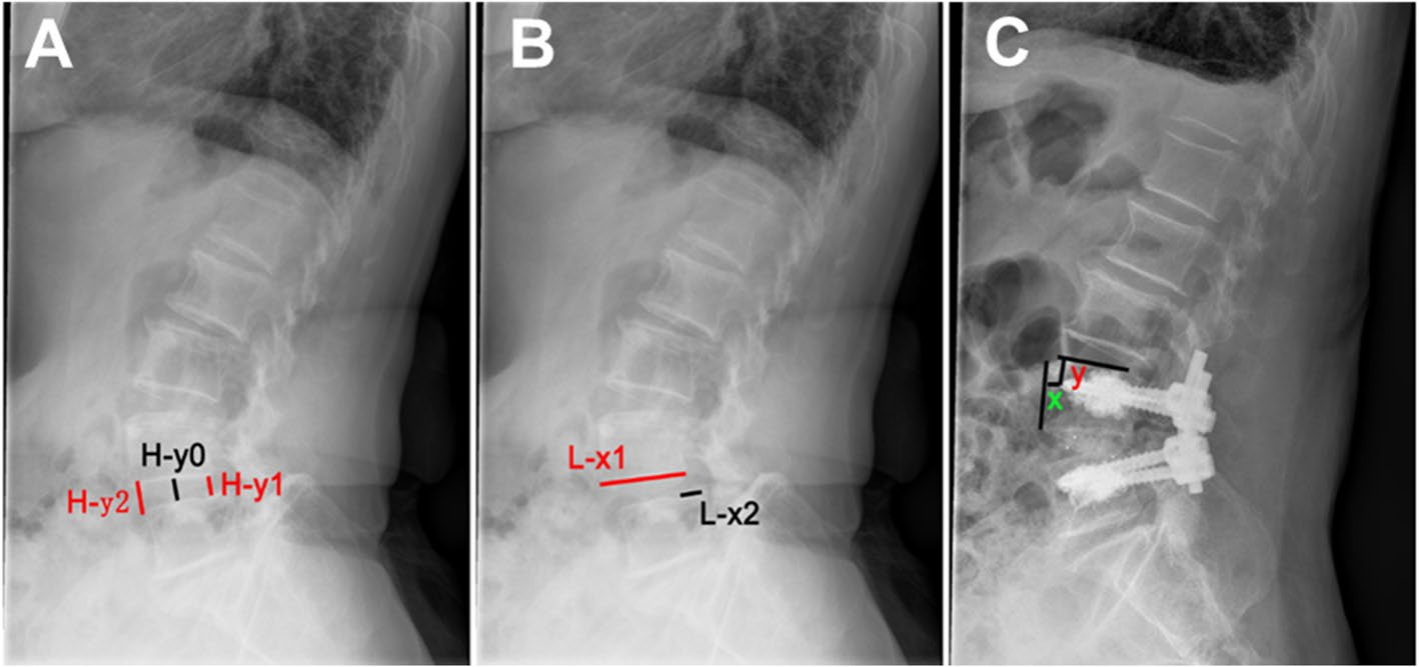
CICPS efficacy evaluation. **A** Height measurement of intervertebral space. **B** Spondylolisthesis degree measurement. **C** CICPSs displacement degree measurement

The efficacy of CICPS was assessed via comparison between preoperative and postoperative VAS and ODI scores at 1, 3, 6, and 12 months and each year thereafter. As the compliance of patients varied, some patients may not follow-up accurately according to the suggested time points, but all the final evaluation were performed more than one-year post operation. The stability of the CICPS was related to the intervertebral height and the Taillard index at the last follow-up. The intervertebral height between the posterior margin of the spondylolisthesis and the upper endplate of the lower vertebra was denoted by H-y1, and the vertical distance between the anterior edge of the lower vertebra and the lower endplate of the spondylolisthesis was denoted by H-y2. The average value of the two was denoted by H-y0 (Fig. 5A). For the Taillard index, we measured the horizontal distance L- × 2 of the upper vertebral body relative to the lower vertebral body slippage and the width L- × 1 of the upper endplate of the lower vertebral body (Fig. 5B). The ratio of the former to the latter is denoted by L- × 0.

The criterion for successful fusion was the presence of a continuous trabecular passage through the fusion zone on the lumbar vertebra clearance [8]. The stability of the CICPS was evaluated by the changes in the distance (x) of the screw tip from the anterior edge of the vertebral body and the distance (y) (Fig. 5C) of the lumbar lateral screw tip from the endplate of the vertebral body after surgery and at the last follow-up. The criterion for CICPS loosening was as follows: [1] lateral CICPS displacement greater than 1 mm [9, 10] and [2] the appearance of a double ring sign around the CICPS [11]. Intraoperative bone cement leakage was classified as described by Yeom [12].

All the imaging data were measured by three experienced spinal surgeons on the INFINITT PACS System (INFINITT Healthcare Co., Ltd, Hangzhou, China), and the mean value was taken. All measurement data were recorded as the mean ± standard deviation. As the statistical indicators follow normal distribution, a paired T test was used to analyze the differences between key statistical indicators (VAS, ODI, H-y0, L- × 0, X, and Y), and data processing was completed with the IBM SPSS 25 version.

## Results

### Basic description of the surgery

All patients had lumbosacral pain of varying degrees, accompanied or not by pain and numbness in one or both lower limbs. Positive and lateral dynamic X-ray, CT and Magnetic Resonance Imaging (MRI) examination showed lumbar spondylolisthesis of different degrees of severity, accompanied by different degrees of severity of intervertebral disc herniation and spinal canal stenosis. All patients underwent conservative treatment for more than half a year without obvious improvement of symptoms. As shown in Table 1, the mean age of the patients was 60.11 ± 7.83 years old, the dual-energy X-ray bone density was -3.16 ± 0.59, the operative time was 223 ± 51 min, the intraoperative blood loss was 427 ± 277 ml, and the mean postoperative hospital stay was 6.2 ± 2.8 days. All the 37 patients were followed up, with an average follow-up time of 26.6 ± 13.4 months. A total of 124 CICPS and 36 pedicle screws were implanted in 37 patients, and 3 of the CICPSs were stopped due to bone cement leakage.

## Effectiveness assessment of CICPS

**Fig. 6:**
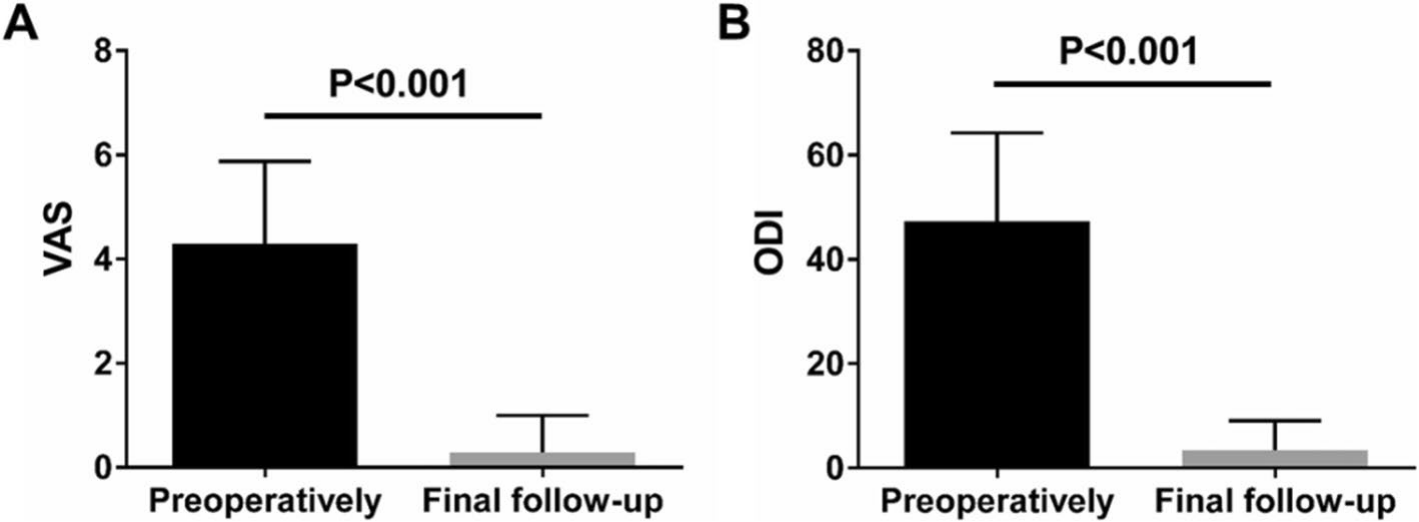
Effectiveness assessment of CICPS. **A** VAS score before operation and at the last follow-up; **B** Preoperative ODI score and the last follow-up ODI score

The 37 patients had varying degrees of relief or even disappearance of symptoms after surgical treatment, and none of them had postoperative deterioration dysfunction or increase in pain relative to the preoperative state. During the follow-up, the VAS score of the patients was significantly decreased from 4.30 ± 1.58 before the surgery to 0.30 ± 0.70 (*P* < 0.001) at the last follow-up (Fig. 6A). The ODI index decreased from 47.27% ± 16.97% before surgery to 3.36% ± 5.70% at the last follow-up (*P* < 0.001) (Fig. 6B). Suggesting the clinical benefit of CICPS usage was definite.

### Stability assessment of CICPS

**Fig. 7:**
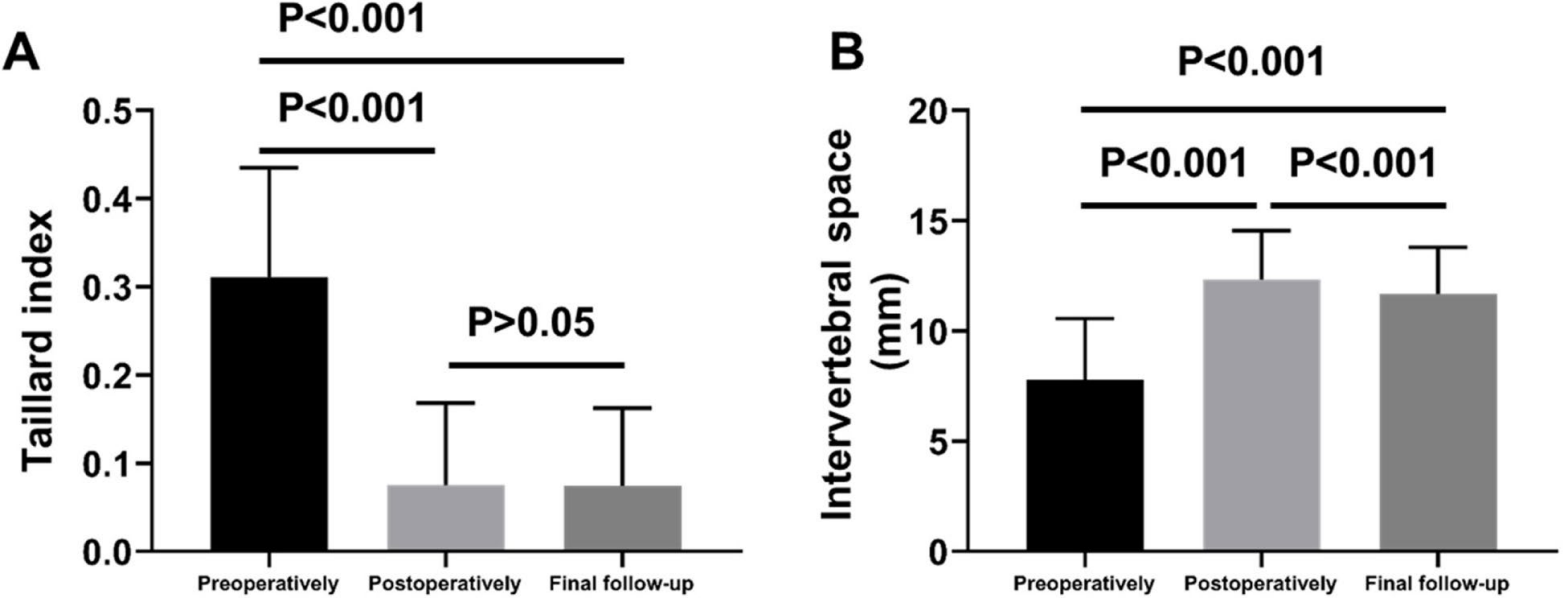
Stability assessment of CICPS. **A** Taillard index changes before surgery, one week after surgery and during the last follow-up; **B** Changes of intervertebral space height before surgery, one week after surgery and at the last follow-up

The preoperative Taillard index L- × 0 of the patients decreased from 0.31 ± 0.02 to 0.07 ± 0.02 (*P* < 0.001) after surgery and to 0.07 ± 0.01 at the last follow-up (*P* < 0.001 compared with preoperative (Fig. 7A), indicating the intraoperative lifting and reduction effect of CICPS was efficient. No vertebral re-slip was observed at the last follow-up, and subsequently, the orthopaedic effect was stable.

The height of intervertebral space H-y0 increased from 7.78 ± 0.56 mm preoperatively to 12.33 ± 0.36 mm postoperatively (*P* < 0.001) and to 11.66 ± 0.35 mm at the last follow-up (*P* < 0.001) (Fig. 7B). Besides, positive and lateral radiographs of the lumbar spine showed continuous trabecular bone passing in all fusion segments, indicating successful fusion.

**Fig. 8:**
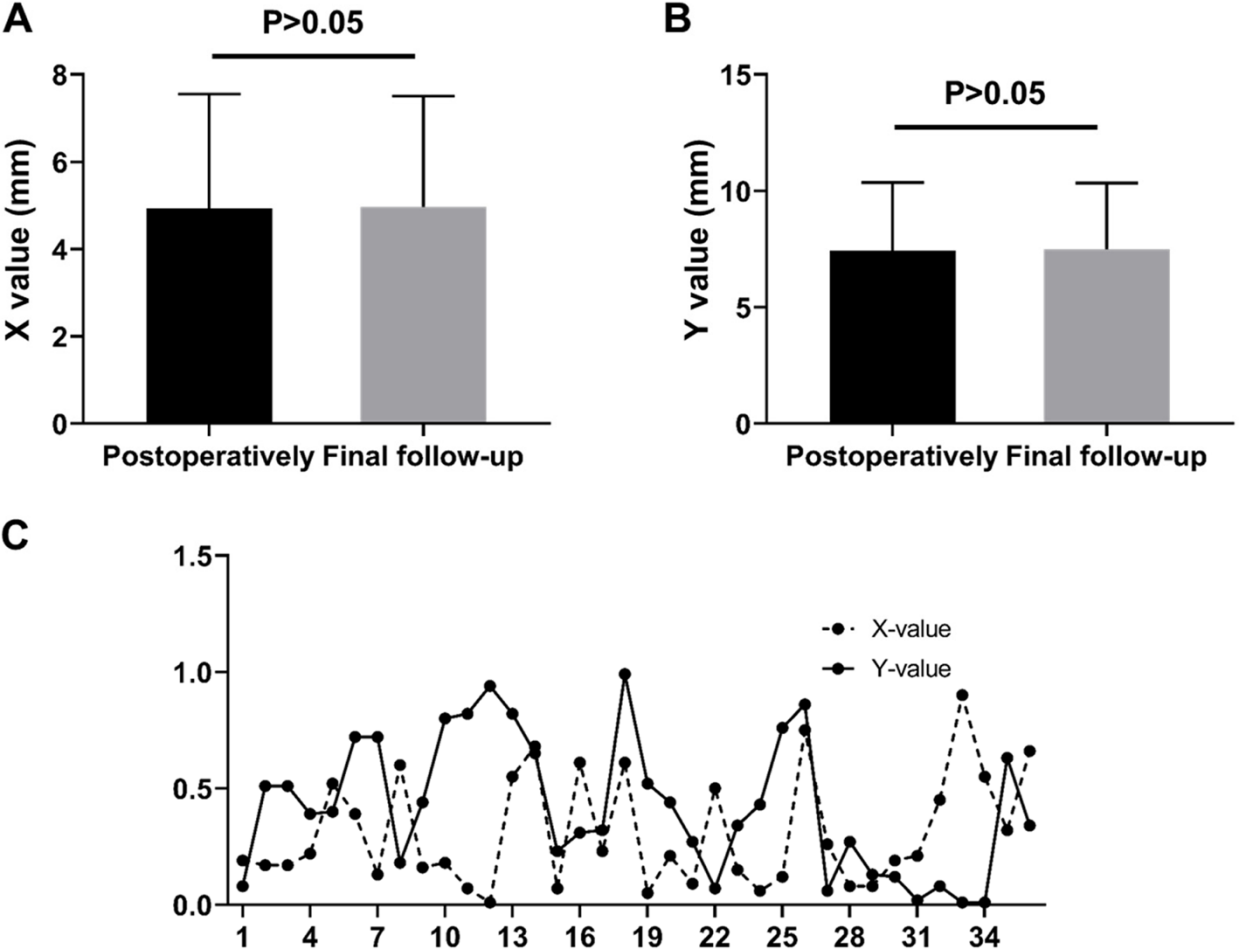
Stability assessment of CICPS. **A** The horizontal distance of CICPS from the apex to the anterior margin of the vertebra (X) after surgery and at final follow-up; **B** The vertical distance of CICPS from the apex to the superior endplate(Y) after surgery and at final follow-up; **C** Represents the absolute difference of *x* value and *y* value between the postoperative and the last follow-up

The postoperative *X* value of the patient was 4.92 ± 0.43 mm and at the last follow-up was 4.97 ± 0.42 mm (*P* = 0.563, Fig. 8A). Postoperative *Y* value was 7.44 ± 0.48 mm and at the last follow-up was 7.48 ± 0.48 mm (*P* = 0.657, Fig. 8B). The absolute difference between the postoperative measured values of X and Y and the final follow-up values of X and Y in all patients was less than 1 mm (Fig. 8C). No obvious double ring sign was observed around CICPS on the lumbar spine frontal radiography, indicating that the CICPS were not loosening or pulling out.

### Complications

Intraoperative vertebral leakage occurred in 3 screws (2.42%), all of which were Yeom type S screws [12]. No symptomatic bone cement pulmonary embolism, deep vein thrombosis occurred after the operation. One patient suffered from a surgical incision infection, which was improved after antibiotic treatment.

## Discussion

Spondylolysis-type lumbar spondylolisthesis occurs when facet joints lose their ability to prevent the upper vertebral bodies from sliding forward. Which leads to the increase of the burden of supraspinal ligaments and interspinous ligaments, and the acceleration of spinal degeneration. Finally, the function of the stable structure between vertebral bodies will lost, and vertebral slip can progress over the III degrees. Up to now, pedicle screw system is still the best surgical method to reconstruct the displaced vertebral body and perform bone fusion for patients with lumbar spondylolisthesis. However, the stability of pedicle screw would be reduced in osteoporosis vertebral bodies, as the thickness of trabeculae and bone cortex decrease and makes screws easy to loosen and pull out. Studies reported that pedicle screw stability is often inadequate for patients with bone mineral density (BMD) below 80 mg/cm^3^ [13], and the screw loosening rate in the osteoporotic vertebral body is up to 60% [14].

To improve the biomechanical strength of pedicle screw in osteoporotic vertebral body, studies have been conducted on adjusting the length, diameter, thread design and reinforcement material of pedicle screws [15–20]. Due to the limited size of pedicles and the presence of osteoporosis, most of the pedicle screws failed to achieve the desired effect. Recently, some studies reported that inflatable pedicle screws [21, 22] and bone cement screws [23, 24]achieved good internal fixation results in patients with osteoporosis associated spinal diseases. However, the efficacy of above-mentioned strategies was mostly limited to patients with mild osteoporosis (Meyerding classification of spondylolysis, level I and II), as cases of vertebral spondylolisthesis above level III were explicitly exclude [22, 23]. Hence, we focused on spondylolysis-type lumbar spondylolisthesis, which is more specific in this study.

Among the various strengthening measures for pedicle screws, PMMA was considered as the best choice owning to its high fixation strength and rapidly coagulation property [4, 25, 26], which meets the demands of intraoperative use of pedicle screws for lifting and reduction of the spondylolisthesis. For the design of bone cement-injectable screws, it is suggested that there would be higher bone cement leakage risk if the proximal side hole of the screw was closer to the nail head [27]. Hence, to improve the fixation strength and reduce the risk of bone cement leakage, the CICPS in this study were designed with three side holes in the front 2/5 of the screw which distributed longitudinally from small to large. Apart from 10 level I and 20 level II patients, we also proved the feasibility of CICPS in 6 level III cases and 1 level IV case (Table 1). No screw fracture was found in any of the follow-up cases, and the bone cement leakage rate was 2.42%, lower than that of similar types of injectable bone cement screws (5%-62.3%) [28], which greatly reduced the possibility of nerve injury.

Fatal pulmonary embolism caused by intraoperative using bone cement screws has been another problem. Insa Jansen [29]and Martin-Fernandez [30] analyzed 1330 and 1780 bone cement screws respectively, and found that most cases of pulmonary embolism occurred in thoracic or thoracolumbar internal fixation and that there was no pulmonary embolism or suspected cases of pulmonary embolism when simply using bone cement screws in lumbar vertebra. In this study, to reduce the risk of pulmonary embolism, only a small amount of bone cement (1–2 ml) was injected into each screw to achieve the fixation strength. As the results proved, the application of bone cement screws in the lumbosacral region alone would not cause pulmonary embolism, which further confirmed the safety of CICPSs in the treatment of lumbar spondylolisthesis (spondylolysis-type) with osteoporosis.

Because of the absence of revision cases in this study, the operability of removing revision screws after surgery is not yet known. After comparing general pedicle screws enhanced by PMMA with hollow side hole cement screws, CHAO et al. found no significant difference in pulling out the screws [31]. M. Martin-Fernandez also declared success in pulling out 180 bone cement reinforced injectable cement screws in various clinical applications [32].

The fusion rate is an important index of internal fixation [33]. In the imaging follow-up of the present study, postoperative L- × 0 was 0.07 ± 0.02 mm, close to final follow-up L- × 0 0.07 ± 0.01 mm (*P* > 0.05). The absolute difference between X and Y was less than 1 mm at the last follow-up, and there was no obvious double ring sign around CICPSs in the lumbar frontal radiography. All the above results indicated that no re-slip of the reduced vertebral body was observed, and no loosening or pulling out of CICPSs was observed.

Although postoperative loss of intervertebral height occurred, the difference was not statistically significant (Fig. 7B). We believe that there are two main reasons for postoperative intervertebral height loss. Firstly, patients are commonly complicated with osteoporosis, and the resistance of bone to pressure is relatively weakened. Studies demonstrated that osteoporosis is an important risk factor for the sinking of the interbody fusion apparatus [34–36]. Secondly, due to the incomplete reduction of the spondylolisthesis during the operation, the biomechanics of the spine changed and the local pressure increased, which also led to the loss of the postoperative intervertebral height. Although no sinking of the cage has been observed due to the relatively small number of cases in this study, the surgeon should still be vigilant for possible sinking of the cage.

This study has several limitations. Firstly, the sample size is small and the follow-up time is not long enough that longer follow-up duration with more cases is needed. Secondly, due to ethical limitations, here only clinical evaluation was performed. A controlled randomized study could be suggested for further study. Finally, standard operating procedure for patients of different degrees was not established, which will be our future research focus.

## Conclusion

In this study, the clinical efficacy and safety of CICPS in the treatment of spondylolysis-type lumbar spondylolisthesis with osteoporosis was investigated, within an average follow-up time of 26.6 ± 13.4 months. Only 3 out of 124 CICPS (2.42%) had bone cement leakage, and no clinical discomfort was found in any patients. Both the VAS score and the ODI decreased significantly preoperative to postoperative. Indicating, CICPSs are safe and effective in the treatment of spondylolisthesis with osteoporosis.

## Data Availability

The datasets used and/or analysed during the current study are available from the corresponding author on reasonable request.

## Abbreviations

CICPS: Cement-injectable cannulated pedicle screw
VAS: Visual Analogue Scale
ODI: Oswestry Disability Index
CPS: Cannulated pedicle screw
PMMA: Poly-methyl-methacrylate
CT: Computed Tomography
MRI: Magnetic Resonance Imaging
BMD: Bone mineral density
